# Survival Outcomes of Esophageal Squamous Cell Carcinoma Patients Who Underwent Salvage Esophagectomy: A Literature Review and Results From Two High‐Volume Centers

**DOI:** 10.1002/ags3.70028

**Published:** 2025-04-29

**Authors:** Kotaro Sugawara, Koichi Yagi, Takashi Fukuda, Shoh Yajima, Daiji Oka, Yoshiyuki Miwa, Shuichiro Oya, Asami Okamoto, Raito Asaoka, Yoshifumi Baba

**Affiliations:** ^1^ Department of Gastrointestinal Surgery, Graduate School of Medicine The University of Tokyo Tokyo Japan; ^2^ Department of Gastroenterological Surgery Saitama Cancer Center Hospital Saitama Japan

**Keywords:** definitive chemoradiotherapy, esophageal squamous cell carcinoma, locoregional recurrence, lymph node dissection, salvage esophagectomy

## Abstract

**Background:**

This study aimed to investigate survival outcomes, the efficacy of lymph node (LN) dissection, and recurrence patterns in patients who underwent salvage surgery (SALV) for esophageal squamous cell carcinoma (ESCC) after definitive chemoradiotherapy (dCRT).

**Methods:**

We retrospectively reviewed 69 patients with clinical stage I–IV thoracic ESCC who underwent SALV. Recurrence patterns and the distribution of LN metastases were analyzed according to the primary tumor location.

**Results:**

The 90‐day mortality rate was 2.9%, and the 3‐year overall survival (OS) rate of the 69 patients was 47.1%. OS curves were significantly stratified by the presence of abdominal LN metastases (*p* = 0.007). Among six patients whose clinically positive LNs were not dissected because their swelling disappeared after dCRT (cN+/CRT‐cN0 cases), two (33.3%) developed locoregional recurrence. In contrast, among 25 patients whose clinically positive LNs were dissected regardless of CRT‐cN status, the incidence of locoregional recurrence alone was 4.0%. Patients with lower thoracic (Lt) tumors had a higher incidence of distant metastases than those with middle (Mt) or upper thoracic (Ut) tumors (61.5% vs. 36.8%/33.3%). Mediastinal LN metastases were rare (7.7%) in Lt tumors, whereas LN metastases were widely distributed within the regional zones in Mt/Ut tumors. Patients with Lt tumors and pathological LN metastases had extremely poor OS (3‐year OS: 0%).

**Conclusions:**

Abdominal LN metastases had a negative impact on survival in ESCC patients who underwent SALV. Clinically positive LNs should be dissected, provided it is technically feasible. The tumor location might influence the distribution and prognostic impact of pathological LN metastases.

## Graphical abstract

This study aimed to investigate survival outcomes, the efficacy of lymph node (LN) dissection, and recurrence patterns in 69 patients who underwent R0 salvage surgery for esophageal squamous cell carcinoma. Of note, abdominal LN metastases had a negative impact on survival in our series. Tumor location significantly influenced the distribution and survival impact of pathological LN metastases.

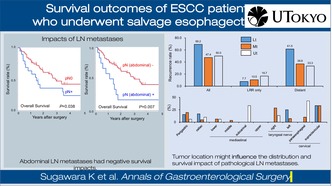

**Abbreviations**
5‐FU: 5‐fluorouracil
CCI: charlson comorbidity index
C‐D: Clavien‐Dindo
CDDP: cisplatin
CF: CDDP + 5‐FU
CI: confidence intervals
CR: complete response
CT: computed tomography
dCRT: definitive chemoradiotherapy
EC: esophageal carcinoma
ESCC: esophageal squamous cell carcinoma
LN: lymph node
LRR: locoregional recurrence
Lt: lower thoracic
MIE: minimally invasive surgery
Mt.: middle thoracic
NDP: nedaplatin
OS: overall survival
PET‐CT: positron emission tomography
PR: partial response
SALV: salvage surgery
Ut: upper thoracic

## 1 Introduction

Esophageal cancer (EC) is the seventh leading cause of cancer‐related mortality worldwide, with esophageal squamous cell carcinoma (ESCC) being the predominant histological subtype in East Asia [1]. Definitive chemoradiotherapy (dCRT) is a primary treatment option for patients with resectable (cStage I–III) ESCC who prefer esophageal preservation, as well as for those with locally advanced, unresectable disease [2].

Clinical trials in Japan have reported that dCRT achieves a clinical complete response (cCR) in approximately 90% of patients with cStage I disease [3], 60% of those with cStage II/III [4, 5], and 15%–33% of individuals with unresectable ESCC [6, 7]. However, disease persistence or recurrence occurs in 40%–75% of cases, posing a significant clinical challenge [4, 8, 9]. In such cases, salvage esophagectomy (SALV) is a crucial intervention that may provide long‐term survival benefits for patients with residual or recurrent disease [10, 11, 12].

Despite its potential advantages, SALV is a highly complex and invasive procedure due to radiation‐induced fibrosis [8, 9, 13], which makes surgical dissection technically challenging. Furthermore, it carries a substantial risk of severe postoperative complications, including anastomotic leakage and tracheal necrosis [10, 11, 12]. Table 1 summarizes key findings from previous studies on SALV outcomes. While some institutions have reported significant survival benefits with this approach, identifying suitable candidates remains a major challenge [10].

**TABLE 1:** Summary of studies on esophagectomy after dCRT.

First author	Year	Study interval	*N*	Residual/recurrence	cT4 (%) before dCRT	Approach OE/MIE	LND	R0 (%)	Morbidity (%)				Mortality (%)	OS (%)			MST (month)	Favorable factors for OS (multivariate)
									Leakage	Pneumonia	RLN palsy	≧ CD3a		1‐Year	3‐Year	5‐Year		
Abe	2024	2013–2022	31	16/15	26.0	28/3	Selective	87.0	16.0	16.0	6.0	74.0^h^	3.0	—	73.0	—	—	NA
Visser	2024	2012–2022	251	—	15.2	72/179	NA	86.0	25.0	23.0	—	43.0	11.0	—	—	—	—	NA
Jie Pan	2023	2018–2021	41	—	20.0	8/33	Radical	76.0	7.0	7.0	24.0	5.0	5.0	63.0	—	—	—	NA
Boerner	2023	2001–2019	173	112/61	2.0	NA	NA	90.0	10.0	27.0	—	33.0	9.3	—	—	26.5	—	NA
Miyata	2022	2002–2017	227	116/111	56.0	NA	Radical	89.0	19.0	18.0	11.0	49.0	5.7	3–year^i^; 33.7/48.7 5–year; 28.0/41.7			—	pT, pN, R0
Mayanagi	2022	2004–2020	52	NA	40.0	52/0	Selective	92.0	27.0	8.0	2.0	64.0	0	—	46.3	—	—	NA
Ishiyama	2022	2007–2020	82	40/42	NA	62/20	Selective	74.4	28.0	41.4	17.1	71.9	2.4	—	—	—	—	NA
Mitchell	2020	2004–2016	35	—	—	31/4	NA	91.4	17.1	34.3	—	54.3	17.1	68.6	45.7	24.2	29.6	NA
Harada	2020	2009–2016	15	7/8	13.3	13/2	Selective	73.3	26.7	0.0	13.3	66.7	0	—	—	—	—	ycN
Sugawara	2020	2006–2016	31	31/0	100.0	73/0	Selective	71.0	16.1	29.0	—	29.0	9.7	—	—	—	—	NA
Sugimura	2020	1997–2017	73	40/33	47.9	31/0	Radical	86.3	19.2	13.7	8.2	46.6	6.8	72.1	44.0	42.0	—	R0, pT0–2, complication
Booka	2020	2004–2016	18	15/3	100.0	18/0	Selective	77.8	38.9	5.6	44.4	—	0.0	88.9	—	51.6	90.1	NA (univariate R0, pneumonia)
Okamura	2020	1998–2016	35^a^	34/1	100.0	NA	Selective	54.3	14.3	28.6	14.3	22.9^f^	8.6	45.7	—	5.7	8.7	R0, pneumonia
Ohkura	2019	2006–2018	33	21/12	100.0	20/3	Radical	42.4	12.1	—	—	33.3	0	—	—	—	—	(DSS; R0, cT4a)
Takeuchi	2018	1994–2017	49	29/20	—	46/3	NA	75.5	16.3	32.7	10.2	—	10.2	—	—	—	24.0	R0, pneumonia, pStage I
Taniyama	2018	2001–2016	100	52/48	8.0	5/95	NA	82.0	25.0	23.0	32.0	76^g^	4.0	—	—	—	—	NA
Kiyozumi	2018	2005–2016	50	32/18	—	50/0	Selective	82.0	20.0	14	—	24.0^f^	0	—	—	—	—	R0, CDIIIa≦, ypStage0–II
Nakajima	2018	2010–2016	16	—	—	10/6	NA	—	6.3	12.5	—	18.8	0.0	—	—	—	—	NA
Sugawara	2018	2006–2016	47^b^	34/13	53.2	47/0	Selective	74.5	29.8	31.9	—	—	10.6	70.0	31.7	—	18.0	R0, CR, GPS 0
Hayami	2017	1988–2015	70	46/24	35.7	67/3	Radical	72.9	12.9	32.9	5.7	60^h^	—	—	—	—	—	R0, < 60 Gy, CR, ypStage 0–II, pulmonary complication
Lertbutsayanukul	2017	2006–2015	44	—	13.6	NA	NA	70.5	6.8	11.4	—	13.6	2.3	—	—	—	25.6	R0, > 60 Gy
Farinella	2016	2006–2014	16^c^	—	18.8	16/0	Radical	81.3	25.0	37.5	—	—	0.0	84.0	63.0	—	—	NA
Watanabe	2015	1988–2013	63	43/20	33.3	60/3	NA	73.0	15.9	36.5	6.3	44.4	7.9	—	29.8	15.0	—	R0, ypT1–2
Matono	2014	1986–2011	20	20/5	50.0	NA	NA	40.0	—	—	—	—	—	—	—	—	—	NA
Chen	2014	1996–2005	51	0/51	—	NA	NA	80.4	5.9	—	—	—	2.0	—	—	—	—	NA
Wang	2014	1999–2012	104	66/38	—	NA	NA	79.8	—	—	—	—	0.0	74.4	39.8	29.5	—	R0, recurrence, LN > 15^e^
Morita	2011	1994–2009	27	7/18	—	25/0	Selective	70.4	37.0	29.6^d^	—	—	7.4	70.2	50.6	50.6	—	R0
Takeuchi	2010	1994–2008	25	—	68.0	25/0	Radical	80.0	24.0	44.0	—	—	8.0	—	—	43	—	R0, bacteremia/sepsis
Tachimori	2009	2000–2006	59	36/23	10.2	59/0	Radical	84.7	30.5	10.1	19.0	—	8.5	—	37.8	—	—	pT1–3, pM0
Miyata	2009	1994–2007	33	13/20	36.4	33/0	NA	87.9	39.4	30.3	27.3	—	12.1	—	—	35	—	NA

Abbreviations: −, data not described; dCRT, definitive chemoradiotherapy; LND, lymph node dissection; MIE, minimally invasive esophagectomy; MST, mean survival time; NA, data not analyzed; OE, open esophagectomy; OS, overall survival; RLN, recurrent laryngeal palsy.

^a^ Included 28 dCRT and 7 RT cases.

^b^ Included 1 adenocarcinoma patient.

^c^ Included 2 adenocarcinoma patients.

^d^ Pneumonia, atelectasis, and hypoxia required reintubation.

^e^ LN > 15 total mediastinal dissection with 15 or more dissected lymph nodes.

^f^ ≧ CD3b.

^g^ Defined according to the Esophageal Complications Consensus Group definitions.

^h^ ≧ CD2.

^i^ Residual/recurrence disease.

Given the limited number of studies on SALV, several unresolved issues remain, including factors influencing survival outcomes, the role of prophylactic lymph node dissection, and patterns of recurrence after surgery [11, 13]. This study aims to address these concerns by evaluating survival outcomes, the impact of lymph node dissection, and recurrence characteristics in ESCC patients undergoing SALV at two high‐volume esophagectomy centers.

## 2 Patients and Methods

### 2.1 Patients

A retrospective analysis was conducted on patients with clinical stage I–IV thoracic ESCC who underwent salvage esophagectomy (SALV) following definitive chemoradiotherapy (dCRT) (radiation dose > 50 Gy, chemotherapy dose intensity > 50%) between 2010 and 2021 at two high‐volume (> 40 annual esophagectomies) esophagectomy centers: the University of Tokyo Hospital and Saitama Cancer Center. Patients who underwent non‐curative (R1/R2) SALV were excluded from the analysis. Treatment strategies were determined through biweekly multidisciplinary cancer board meetings, which included radiologists, gastroenterologists, and surgeons who collaboratively discussed each case. Clinical data were retrospectively extracted from a prospectively maintained database. Additionally, comorbidities were categorized using the Charlson Comorbidity Index (CCI). At the time of the final follow‐up (October 2024), the median follow‐up period was 80.4 months for the survivors. This retrospective study was approved by the local ethics committee of each hospital (ID: 3962, 1250, 1604).

### 2.2 Clinical Staging

Before treatment, the clinical stage was determined by the Multidisciplinary Tumor Board based on the results of esophagogastroduodenoscopy, computed tomography (CT), and/or positron emission tomography (PET‐CT), as described in previous studies [8, 9]. On CT and PET‐CT, round‐shaped lymph nodes with a long axis ≥ 1 cm, or with FDG uptake higher than the background, were generally considered clinically positive. Node zones were designated as described in Table S1. Clinical and histological tumor staging was based on the TNM classification (UICC, 8th edition) [14].

### 2.3 Definitive Chemoradiotherapy and Initial Evaluation

Definitive CRT was performed as previously described [8, 15]. Definitive chemoradiotherapy typically consists of chemotherapy combined with radiation (50.4–60, 1.8–2.0 Gy per day in 28–30 fractions, five times per week) to the primary tumor and any metastases, as well as more than 40 Gy of prophylactic radiation to the regional lymph nodes. At the University of Tokyo Hospital, the chemotherapy regimen primarily consisted of 2–4 courses of chemotherapy combining 5‐fluorouracil (5‐FU) or oral TS‐1 with cisplatin (CDDP) [9]. At the Satima Cancer Center, as the first choice for the dCRT regimen, the standard‐dose CDDP+5‐FU (CF) protocol of JCOG0303 was selected [16]. Additional CF therapy was continued as needed following dCRT. For patients with renal or cardiac dysfunction, nedaplatin (NDP) was used instead of cisplatin (CDDP). After completing dCRT or induction chemotherapy, all patients were restaged using endoscopy, CT, and/or PET‐CT to evaluate the clinical response [17, 18]. All patients undergoing dCRT were evaluated within 2 months after the completion of dCRT.

### 2.4 Treatment Strategy and Surgical Procedure

Patients who achieved a clinical complete response (CR) underwent regular follow‐up monitoring. For those with a clinical partial response (PR) where residual cancer was suspected or confirmed, surgical resection was typically performed within 2–4 months after completing dCRT, provided that curative esophagectomy was considered feasible. Patients with stable or progressive disease received additional chemotherapy or palliative care as appropriate. The basic SALV procedure consisted of subtotal esophagectomy using a cervico‐thoraco‐abdominal approach [9, 15]. For SALV, we generally performed limited lymph node dissection that is, harvesting only clinical node‐positive locations that were swollen or suspected of harboring a recurrence.

The operative thoracic approach was by video‐assisted thoracoscopic surgery or thoracotomy. A gastric conduit was passed through the retrosternal or posterior mediastinal route. Esophagogastric anastomosis was made at the thoracic (for thoracotomy) or neck (for thoracoscopic surgery). The Clavien‐Dindo scale was used to grade all postoperative morbidities.

### 2.5 Post‐Treatment Follow‐Up Evaluation

Tumor response grading for the resected specimen was categorized based on the criteria of the Japanese Esophageal Society [19, 20]. Postoperative surveillance was performed based on the Guidelines for Diagnosis and Treatment of Carcinoma of the Esophagus [21]. Post‐treatment surveillance for cancer recurrence included measuring blood tumor markers (SCC, CYFRA, P53, and CEA) and obtaining CT scans every 3–6 months after the patients had been discharged, and esophagogastroduodenoscopy was performed annually. Positron emission tomography scanning was added for patients in whom recurrence was indicated by other diagnostic modalities.

### 2.6 Definitions of Recurrence

Disease recurrences were classified as solely locoregional recurrence (LRR) or distant recurrence [15]. Patients who experienced recurrence at the anastomotic site or in regional lymph nodes, including those in the cervical (supraclavicular included), mediastinal, or abdominal regions, were classified into the locoregional recurrence (LRR) group. Those with recurrence in distant organs, such as the lungs, brain, or liver, were categorized into the distant recurrence group. Additionally, patients with lymph node recurrence in the paraaortic region were also assigned to this group. If both locoregional and distant recurrences were present, the patient was classified under the distant recurrence group.

### 2.7 Statistical Analysis

Categorical variables were compared using Fisher's exact test or the χ^2^ test, as appropriate. Continuous variables were compared using Wilcoxon's rank‐sum test or ANOVA, as appropriate. Overall survival (OS) was calculated from the start of the initial treatment. Kaplan–Meier survival curves were constructed to estimate survival, and we used the log‐rank test for making comparisons. Statistical analyses were carried out using JMP 18.0.0 (SAS Institute, Cary, NC).

## 3 Results

### 3.1 Patient Characteristics

A total of 69 patients who underwent curative (R0) salvage esophagectomy (SALV) were included in this study. The clinicopathological characteristics of these patients are summarized in Table 2. The mean patient age was 63 years, and the primary tumor was most commonly located in the middle thoracic esophagus (*n* = 38, 55.1%). The distribution of clinical stages was as follows: cStage I (*n* = 5, 7.2%), cStage II (*n* = 11, 15.9%), cStage III (*n* = 24, 34.8%), and cStage IV (*n* = 29, 42.1%). Among the surgical approaches, conventional open transthoracic esophagectomy was performed in 38 patients (55.1%), while 31 patients (44.9%) underwent minimally invasive esophagectomy (MIE). Selective lymphadenectomy was conducted in 52 patients (75.4%).

**TABLE 2:** Patient characteristics.

Variables	No. of patients (%)
Age (years), Mean (range)	63 (47–81)
Sex, male: female	51 (73.9)/18 (26.1)
CCI ≥ 2	11 (15.9)
Main Locus, Ut/Mt/Lt	18 (26.1)/38 (55.1)/13 (18.8)
Residual/Recurrent cancer	42 (60.9)/27 (39.1)
Clinical stage^a^	
cT1/T2/T3/T4	6 (8.7)/6 (8.7)/30 (43.5)/27 (39.1)
cN0/cN1‐2	21 (30.4)/48 (69.6)
cM0/M1	62 (89.9)/7 (10.1)
cStage I/II/III/IV	5 (7.2)/11 (15.9)/24 (34.8)/29 (42.1)
CRT regimen	
Chemotherapy regimen	
CF/NDP + TS‐1/DCF/Others	29 (42.0)/26 (37.6)/7 (10.1)/7 (10.1)
Radiation dose, 50.4Gy/≥ 60 Gy	38 (55.0)/31 (45.0)
Surgical outcomes	
Surgical procedures, OE/MIE	38 (55.1)/31 (44.9)
Lymaphanedectomy, selective/radical	52 (75.4)/17 (24.6)
Morbidity^b^, Grade IIIa/IIIb/IV/V	13 (18.8)/2 (2.9)/3 (4.3)/2 (2.9)
Details of complications (≥ Grade III)^b^	
Anastomotic leakage	10 (14.5)
Pneumonia	8 (11.6)
Chylothorax	2 (2.9)
Pleural effusion	1 (1.4)
Tracheal necrosis	1 (1.4)
Intrathoracic abscess	1 (1.4)
Hiatal hernia	1 (1.4)
Pathological stage^a^	
pT0/T1/T2/T3/T4	24 (34.8)/15 (21.7)/6 (8.7)/24 (34.8)
pN0/N1/N2‐3	50 (72.5)/12 (17.4)/7 (10.1)
pM0/M1	66 (95.7)/3 (4.3)
pStage 0/I/II/III–IV	22 (31.9)/8 (11.6)/24 (34.8)/15 (21.7)

Abbreviations: CCI, charlson comorbidity index; CF, CDDP+5‐FU; CRT, chemoradiotherapy; DCF, docetaxel + CDDP + 5‐FU; MIE, minimally invasive esophagectomy; NDP, nedaplatin; OE, open esophagectomy.

^a^ 8th TNM classification.

^b^ Clavien‐Dindo classification.

Postoperative complications classified as Clavien‐Dindo grade III or higher occurred in 20 patients (29.0%). Details of postoperative complications (≥ Clavien‐Dindo grade III) were also shown in Table 2. The incidence of postoperative complications (CD3 or higher) did not differ significantly between patients who underwent selective lymphadenectomy and patients with radical lymphadenectomy (28.9% vs. 29.4%, *p* = 0.96). Patients who received radical lymphadenectomy developed pneumonia more frequently than patients who underwent selective lymphadenectomy (23.5% vs. 7.7%, *p* = 0.09). The 90‐day mortality rate was 2.9% (*n* = 2). Both patients died from interstitial pneumonia. Pathological complete response (pCR; pStage 0) was observed in 22 patients (31.9%).

### 3.2 Pathological LN Status Was Associated With Overall Survival

**FIGURE 1:**
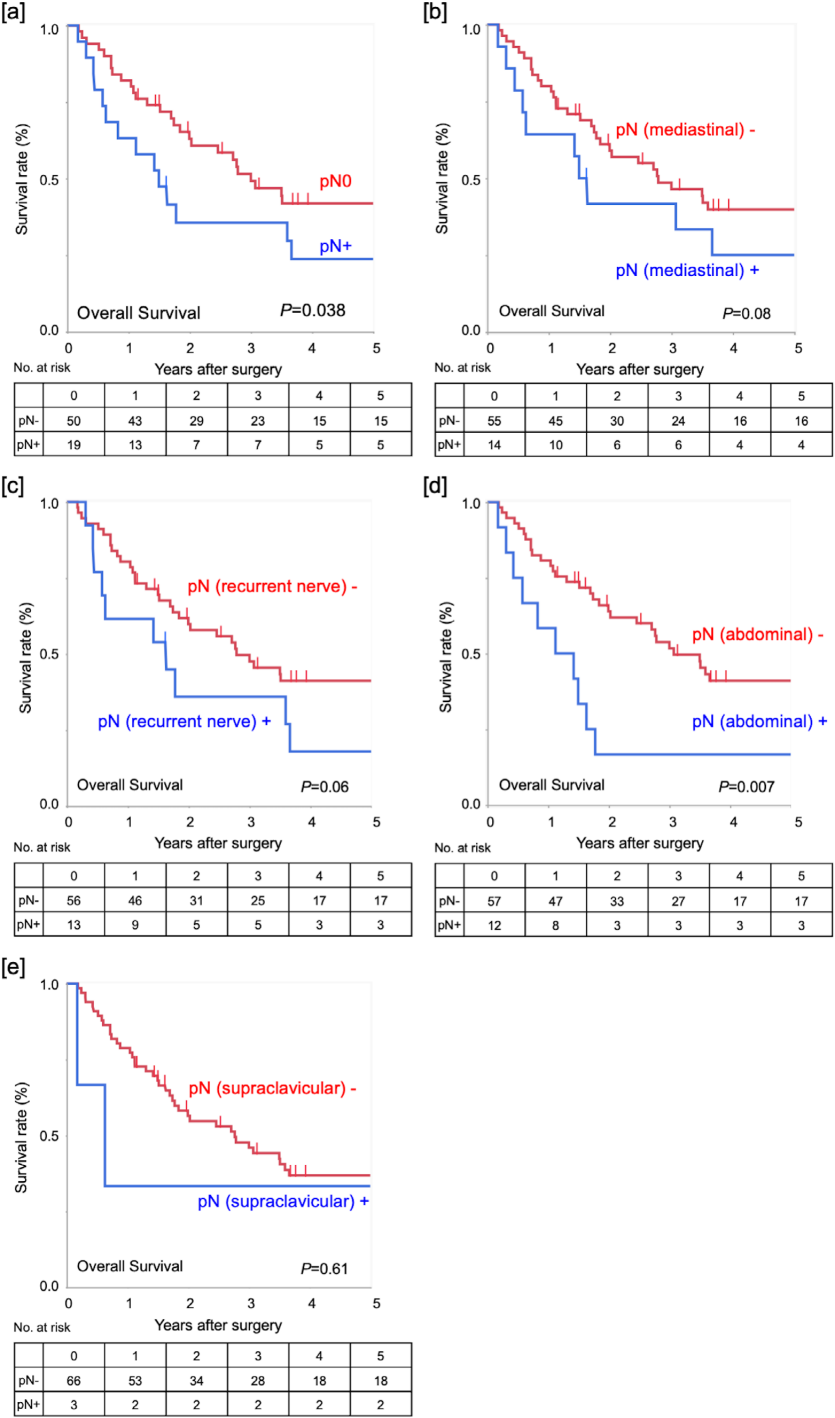
Survival according to pN distribution. OS curves according to the presence of (a) overall pathological LN metastasis, (b) mediastinal LN metastasis, (c) recurrent laryngeal nerve LN metastasis, (d) abdominal LN metastasis, and (e) supraclavicular LN metastasis.

The 1‐ and 3‐year overall survival (OS) rates among the 69 patients were 76.8% and 47.1%, respectively. Univariable analysis using the Cox proportional hazards model identified pathological lymph node (LN) metastasis (HR 1.89, 95% CI 1.03–3.47, *p* = 0.04) and pStage III–IV disease (compared to pStage 0; HR 2.31, 95% CI 1.03–5.19, *p* = 0.04) as significant predictors of poor OS (Table 3). OS curves were significantly demarcated by the presence of pN+ (Figure 1a, *p* = 0.038). The presence of mediastinal or recurrent laryngeal nerve LN metastases did not significantly stratify the OS curves (Figure 1b,c, *p* = 0.08 and 0.06, respectively). Of note, patients with abdominal LN metastases had significantly poor OS than patients without abdominal LN metastases (Figure 1d, *p* = 0.007). The OS of patients with supraclavicular LN metastases was similar to that of patients without supraclavicular LN metastases (Figure 1e, *p* = 0.61). Overall, pathological LN metastases, especially abdominal LN metastases, had negative survival impacts in patients who underwent R0 SALV.

**TABLE 3:** Cox proportional hazards model analysis for overall survival.

Variables	Univariable analysis		
	HR	95% CI	*p*
Preoperative characteristics			
Male	1.52	0.75–3.07	0.24
Age > 65	0.88	0.49–1.59	0.68
CCI ≥ 2	0.69	0.28–1.77	0.45
Tumor location			
Ut	Ref		
Mt.	1.34	0.66–2.70	0.42
Lt	1.36	0.56–3.28	0.49
Clinical stage			
cN+	1.44	0.76–2.74	0.27
cStage III‐IV (vs. I–II)	0.71	0.35–1.39	0.31
MIE (vs. OE)	0.81	0.45–1.48	0.51
Lymphadenectomy			
Radical (vs. selective)	1.02	0.53–1.98	0.96
Complications (C‐D ≥ Grade III)	1.29	0.68–2.46	0.44
Pathological findings			
pT3‐4 (vs. pT1‐2)	1.42	0.79–2.58	0.24
pN+ (vs. pN0)	1.89	1.03–3.47	0.04
pStage			
pStage 0	Ref		
pSt I	1.71	0.62–4.70	0.29
pSt II	1.64	0.76–3.57	0.21
pSt III–IV	2.31	1.03–5.19	0.04

Abbreviations: C‐D, Clavien‐Dindo; CCI, charlson comorbidity index; MIE, minimally invasive esophagectomy; OE, open esophagectomy.

### 3.3 Recurrence Pattern in Patients With Residual Tumors

**FIGURE 2:**
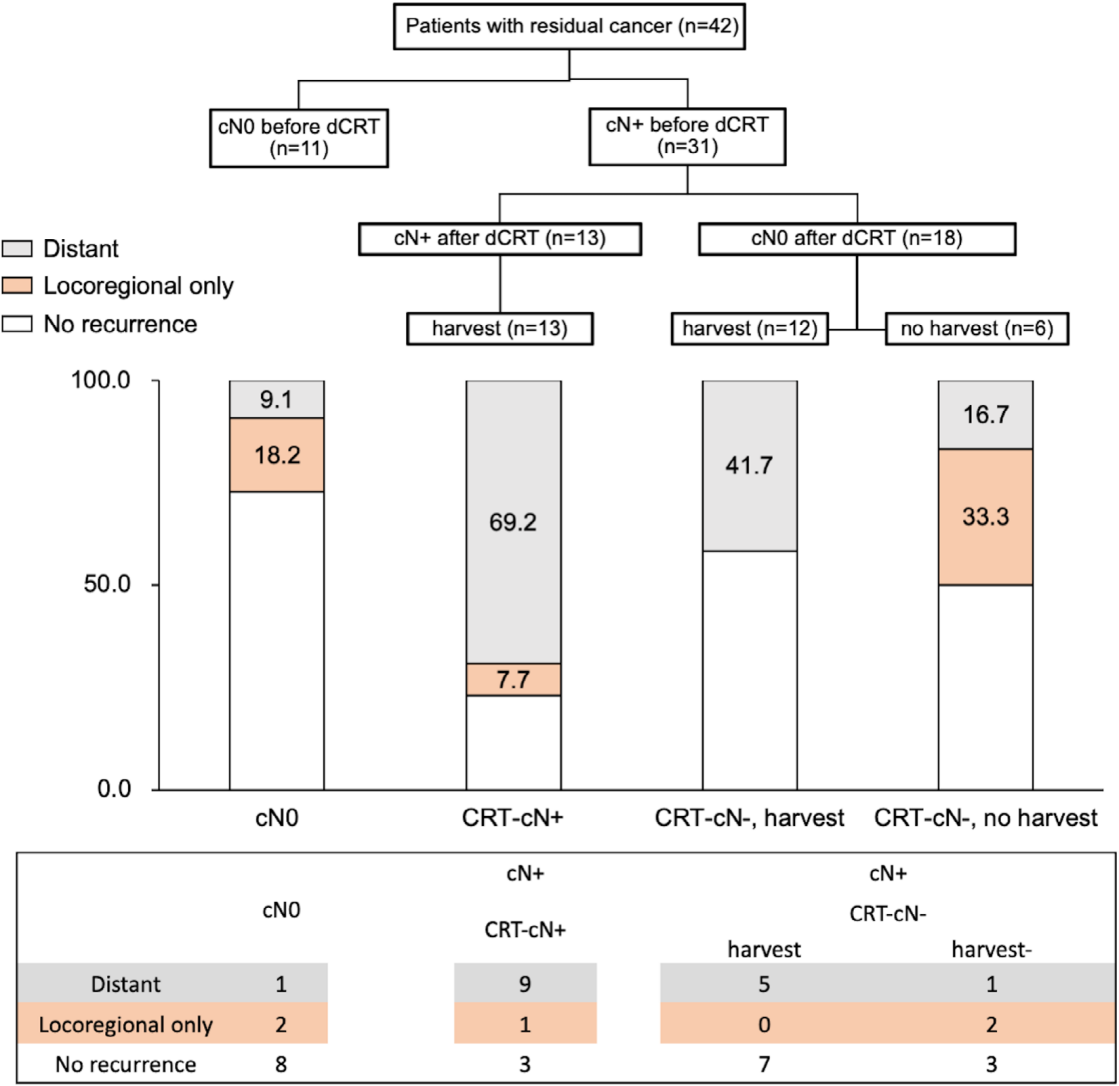
Recurrence pattern in patients with residual tumors. Among the 42 patients with residual cancer after dCRT, 31 had clinically positive LNs before dCRT (cN+), while 11 did not (cN0). Of the six patients whose clinically positive LNs were not dissected because their swelling resolved after dCRT (cN+/CRT‐cN0 cases), two (33.3%) developed locoregional recurrence. In contrast, among the 25 patients whose clinically positive LNs were dissected regardless of their CRT‐cN status, the incidence of locoregional recurrence alone was 4.0% (1/25).

Among the 42 patients with residual cancer after dCRT, 31 had clinically positive lymph nodes (LNs) before treatment (cN+), while 11 did not (cN0) (Figure 2). In 13 patients with residual clinically positive LNs after dCRT (cN+ and CRT‐cN+), all suspected LNs were resected. Among these cases, distant metastases occurred in nine patients (69.2%), while locoregional recurrence (LRR) was observed in only one patient (7.7%) (Figure 2).

In 18 cases, LNs that were enlarged before dCRT and suspected to be metastatic (cN+) regressed after treatment (CRT‐cN0). Of these, six patients did not undergo LN dissection due to technical challenges in preserving critical organ blood flow. In this subgroup, LRR occurred in two patients (33.3%) (Figure 2). In both cases, the primary tumor was located in the upper thoracic (Ut) region, and LRR developed in the upper mediastinum (Figure S1).

In contrast, among 25 patients who underwent LN dissection regardless of CRT‐cN status (CRT‐cN+: *n* = 13, CRT‐cN0: *n* = 12), the incidence of isolated LRR was only 4.0% (1/25) (Figure 2).

### 3.4 Recurrence Pattern According to the Tumor Location

**FIGURE 3:**
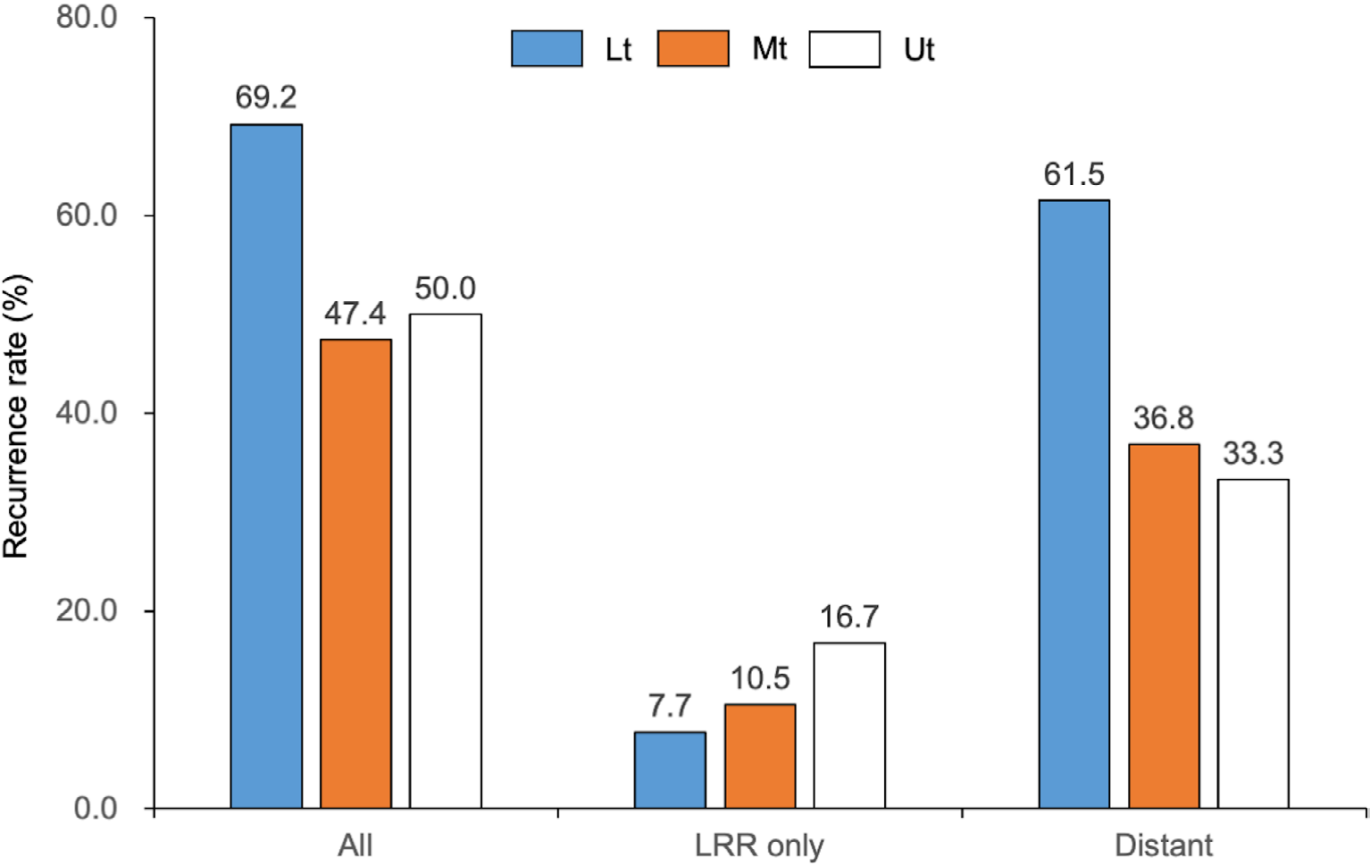
Recurrence rate and pattern according to tumor location. The recurrence developed more frequently in patients who had Lt tumors than those with Mt/Ut tumors (69.2% vs. 47.4/50.0%). Patients with Lt tumors had a high incidence of distant metastases as compared with those with Mt/Ut tumors (61.5% vs. 36.8/33.3%).

Recurrence occurred more frequently in patients with Lt tumors compared to those with Mt/Ut tumors (69.2% vs. 47.4%/50.0%, Figure 3). Notably, the majority of recurrences in patients with Lt tumors were distant metastases (8/9). As a result, patients with Lt tumors exhibited a higher incidence of distant metastases than those with Mt/Ut tumors (61.5% vs. 36.8%/33.3%, Figure 3). The extent of lymphadenectomy did not significantly differ based on tumor location in our cohort (Figure S2).

### 3.5 The Distribution and the Survival Impacts of Lymph Node Metastasis

Table S1 outlines the node zone names used in our study. In patients with Lt tumors, pathological lymph node (LN) metastases were predominantly found in the abdominal and supraclavicular regions. In contrast, mediastinal LN metastases were rare, with only one patient showing metastasis along the left laryngeal nerve (Table 4). Notably, patients with Lt tumors and pathological LN metastases had extremely poor overall survival (3‐year OS; 0%) (Table 4). In patients with Mt/Ut tumors, pathological LN metastases were more widely distributed across the abdominal, mediastinal, and cervical regions (Table 4). Some patients with Mt/Ut tumors exhibited relatively favorable OS, even in the presence of pathological LN metastases (Table 4).

**TABLE 4:** The incidence and the survival impacts of each lymph node metastasis.

	Lt (*n* = 13)			Mt (*n* = 38)			Ut (*n* = 18)		
	Dissection rate (%)	Pathological positive (%)	3 year‐OS (%)	Dissection rate (%)	Pathological positive (%)	3 year‐OS (%)	Dissection rate (%)	Pathological positive (%)	3 year‐OS (%)
Perigastric	100	15.4	0	94.7	19.4	0	94.4	5.9	100
Celiac	46.2	16.7	0	55.3	4.8	100	55.6	10	0
Lower mediastinum	84.6	0		84.2	6.3	0	94.4	5.9	100
Middle mediastinum	92.3	0		73.7	3.6	0	94.4	0	
Subcarnial	53.8	0		47.4	0		16.7	33.3	0
Upper mediastinum	61.5	0		63.2	0		72.2	0	
Right laryngeal nerve	61.5	0		55.3	28.6	44.4	83.3	13.3	50
Left laryngeal nerve	30.8	25	0	36.8	7.1	0	55.6	0	
Cervical paraesophageal	38.5	0		26.3	10	0	38.9	42.9	66.7
Supraclavicular	23.1	33.3	0	15.8	33.3	50	16.7	0	

Abbreviations: Lt, lower thoracic; Mt, middle thoracic; OS, overall survival; Ut, upper thoracic.

## 4 Discussion

As described in Table 1, SALV is reportedly associated with high morbidity and mortality [8, 9, 10]. In our cohort, the overall morbidity (≥ C‐D Grade III) was 29.0%, and the 90‐day mortality rate was 2.9%, both of which were better than previously reported short‐term outcomes (as shown in Table 1). Our cohort consisted of patients treated at two high‐volume esophagectomy centers, which may have contributed to these favorable short‐term outcomes [22]. Nevertheless, the 3‐year OS rate for the 69 patients who underwent curative SALV was 47.1%, underscoring the unsatisfactory treatment outcomes of SALV for ESCC patients.

Strategies to reduce postoperative complications, particularly pulmonary issues, are essential for improving treatment outcomes after SALV. Surgeons often opt for elective lymph node dissection, such as omitting subcarinal lymph node dissection, to preserve blood flow to the trachea [11]. This approach is reportedly useful for reducing fatal tracheal and pulmonary problems in patients who underwent SALV [11, 23, 24]. In fact, our study suggested radical lymphadenectomy to increase the incidence of postoperative pneumonia. However, given the biologically aggressive nature of residual or recurrent ESCC after dCRT, extensive lymph node dissection may offer survival benefits for patients with this tumor type. In fact, some researchers have suggested that prophylactic mediastinal lymph node dissection may provide these benefits [13, 25].

Notably, among the six patients whose clinically positive LNs were not harvested because their swellings resolved after dCRT (cN+ but CRT‐cN0), LRR occurred in two patients (33.3%). Both tumors were located in the upper thoracic region, and LRR developed in the upper mediastinum. In contrast, when clinically positive LNs were dissected, the incidence of LRR was only 4.0%. In some cases, performing LN dissection was technically challenging, particularly in SALV procedures for tumors located in the middle or upper thoracic regions, as these areas are in close proximity to vital structures such as the aorta and trachea. This technical difficulty may explain the higher incidence of LRR in patients with Mt/Ut tumors [13, 25]. Given that the diagnostic accuracy of metastasis LNs during dCRT remains low [11, 26], clinically suspected LN metastasis should be dissected, provided it is technically feasible.

Previous studies have emphasized the significant survival benefit of achieving R0 resection in SALV cases (Table 1), although research specifically focusing on R0 cases remains limited. Our findings indicate that pathological nodal positivity (pN+), particularly abdominal pN+, has a notable survival impact in patients who underwent R0 SALV. Additionally, in our cohort, approximately 76.9% of patients with cN+ and CRT‐cN+ status experienced recurrence. These findings, in conjunction with previous research [10, 13], highlight the crucial role of lymph node status in determining survival outcomes in patients who achieve R0 SALV. The precise mechanisms underlying the oncological impact of abdominal lymph node metastasis remain unknown. A recent study suggested clinical abdominal lymph node metastasis to reflect the potential risk of postoperative systemic recurrence, and is thereby associated with poor oncological outcomes in patients who underwent surgery for ESCC [27].

Only a few studies have examined the recurrence patterns after dCRT. A recent study revealed that LRR was rare in patients who achieved R0 SALV [25], while another found that the recurrence patterns were similar between SALV and non‐SALV cases [13]. In our study, the incidence of LRR was 7.7%, 10.5%, and 16.7% for Lt/Mt/Ut tumors, which appeared to be lower than the previously reported LRR incidence in patients who underwent chemotherapy or CRT followed by surgery [28]. These findings suggest that dCRT provides superior control of local tumors.

It is noteworthy that we found the distribution and survival impacts of pathological LN metastasis to differ greatly between Lt and Mt/Ut tumors, an observation suggesting that tumor biology varies significantly based on tumor location in SALV cases. A nationwide observational study, which investigated a large Japanese cohort mainly comprised of non‐SALV cases, revealed that mediastinal LN metastases were not uncommon in Lt tumors (20%–25%), and patients with Lt tumors and mediastinal LN+ had a moderate 5‐year OS of approximately 30% [29]. On the other hand, previous studies have suggested that recurrence patterns differ greatly between Ut/Mt and Lt tumors in patients undergoing upfront surgery [30], and have shown that the pattern of LN metastasis varies by tumor location in patients who underwent neoadjuvant CRT [31]. These findings suggest the tumor biology to greatly differ according to the primary tumor sites, although studies with larger cohorts comprised of non‐SALV and SALV cases are needed to both confirm and assess our results in further detail.

Overall, the extent of LN dissection and treatment strategy should be tailored based on tumor location in SALV cases; however, LN dissection strategy remains to be optimized. Radical mediastinal LN dissection should be considered for Mt/Ut tumors; however, it is difficult to perform extensive LN dissection while retaining safety. Given the low incidence of mediastinal LN metastasis in Lt tumors, extensive mediastinal LN dissection can be omitted for these tumors.

Our cohort included 22 (31.9%) pStage0 patients. Surgery was considered for these patients based on endoscopy or CT findings: severe stenosis of the esophagus (*n* = 5), ulcer (*n* = 3), irregular surface (*n* = 3), protruded changes (*n* = 2), wall thickening (*n* = 7), and iodine‐unstained areas (*n* = 2). According to the Japanese Classification of Esophageal Cancer, clinical complete response was defined based on both endoscopy/CT findings and histological findings [32]. Reliable diagnostic criteria for clinical response remain to be established in clinical practice [33]. The relatively aggressive strategy deems reasonable for patients with remnant or recurrent ESCC after dCRT [33]. However, prior studies have shown that patients with a good response to preoperative CRT do not gain a survival benefit from subsequent surgical treatment. As such, an active surveillance strategy could be a useful therapeutic alternative for ESCC patients who respond well to chemoradiotherapy [34]. Overall, the benefits of SALV for good responders remain controversial.

Our study showed pN+ to be significantly associated with poor OS, especially in Lt tumors. Accordingly, patients with pN+ may be a good indication for adjuvant therapy after SALV, as indicated in a recent investigation [18]. Adjuvant nivolumab reportedly improved survival in EC patients who undergo curative surgery after preoperative CRT [4]. Additional therapy may be tailored based on the characteristics of the primary tumor lesion.

This study has several limitations. First, although it included a relatively large cohort of ESCC patients who underwent SALV compared to previous studies, it was a retrospective analysis conducted at two institutions. Second, while treatment decisions were made during multidisciplinary team meetings, selection bias may have influenced surgical indications and treatment modalities due to the absence of randomization. Third, the treatment strategies for ESCC have evolved significantly over the course of the study period. Recently, immune checkpoint inhibitors have become a standard treatment for ESCC patients [35]. Finally, around 40% of patients in this study were treated with the NDP and TS‐1 regimen for definitive CRT, whereas the CROSS regimen (paclitaxel and carboplatin) has become widely used in the West, which may have impacted the survival outcomes.

Overall, LN metastasis, particularly abdominal LN metastasis, negatively impacted survival in patients who underwent curative SALV. Clinically positive LNs should be harvested, even if their swelling subsides after dCRT, provided it is technically feasible. Tumor location significantly influenced the distribution and survival impacts of pathological LN metastasis, suggesting the diverse tumor biology associated with different primary tumor sites.

## Author Contributions

**Kotaro Sugawara:** conceptualization, methodology, investigation, writing – original draft, project administration, data curation, software. **Koichi Yagi:** conceptualization, methodology, writing – review and editing. **Takashi Fukuda:** conceptualization, methodology, writing – review and editing. **Shoh Yajima:** data curation, writing – review and editing, supervision. **Daiji Oka:** data curation, writing – review and editing, validation. **Yoshiyuki Miwa:** writing – review and editing, data curation, validation. **Shuichiro Oya:** data curation, writing – review and editing, validation, investigation. **Asami Okamoto:** writing – review and editing, data curation, investigation. **Raito Asaoka:** writing – review and editing, data curation, investigation. **Yoshifumi Baba:** conceptualization, methodology, writing – review and editing, supervision.

## Ethics Statement

All procedures were in accordance with the ethical standards of the responsible committee on human experimentation (institutional and national) and with the Helsinki Declaration of 1964 and later versions.

## Consent

Informed consent was obtained from all individual participants in the form of opt‐out on the website. Those who rejected participation were excluded.

## Conflicts of Interest

Dr. Yoshifumi Baba is an Editorial Board Member of AGS. The other authors have no conflicts of interest to disclose.

## Supporting information


**Figure S1.** Locoregional recurrence in cN+ but CRT‐cN‐ cases.

**Figure S2.** The extent of lymphanedectomy according to tumor location.

**Table S1.** Node zones.
