# Supplementary figures and images for: Survival Outcomes of Esophageal Squamous Cell Carcinoma Patients Who Underwent Salvage Esophagectomy: A Literature Review and Results From Two High‐Volume Centers
Source: Ann Gastroenterol Surg. 2025 Apr 29;9(5):952–63. doi: 10.1002/ags3.70028 (PMC12414584; doi:10.1002/ags3.70028)

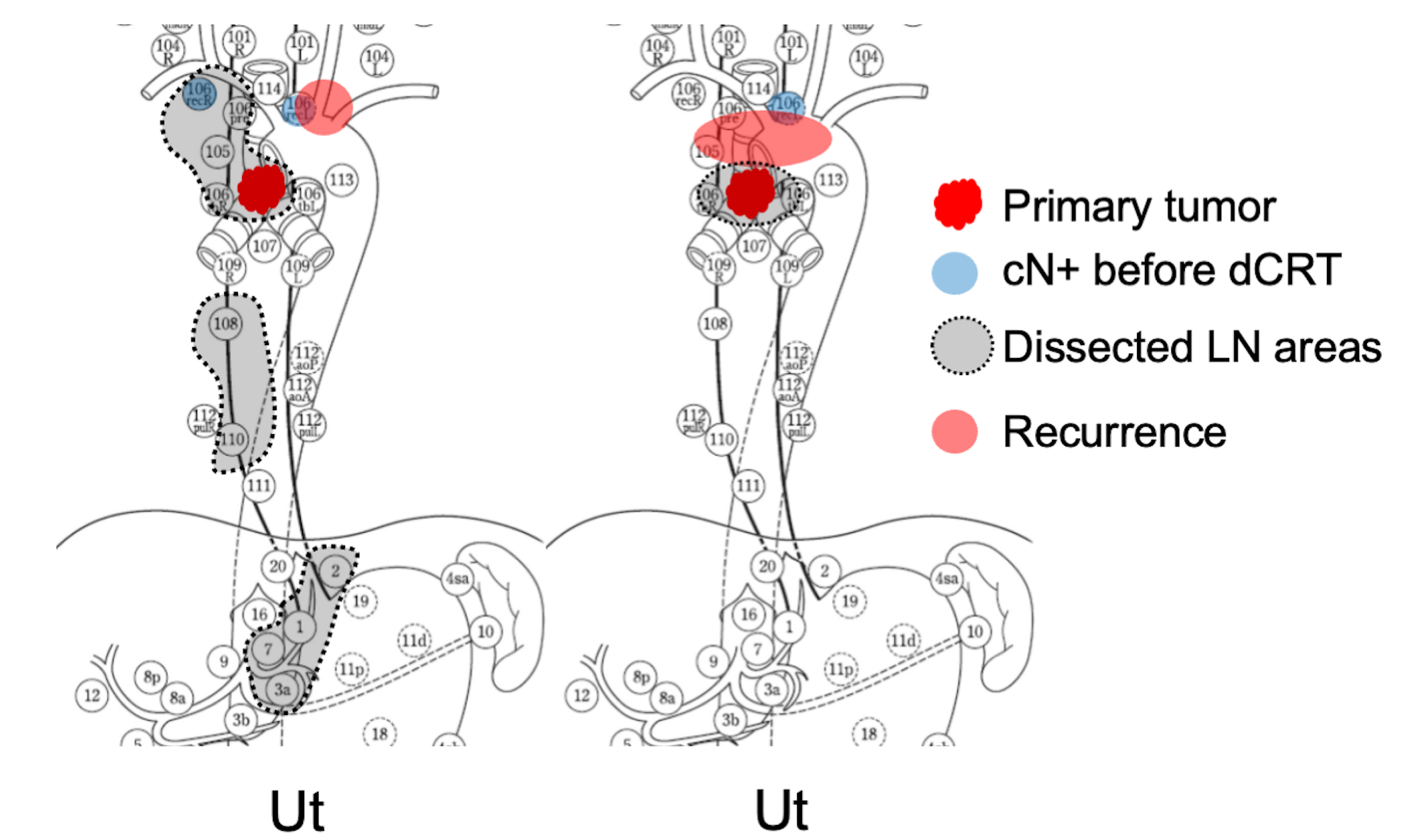

Supplement: Supplementary file 1 — Figure S1. Locoregional recurrence in cN+ but CRT‐cN‐ cases. [file AGS3-9-952-s003.tiff]

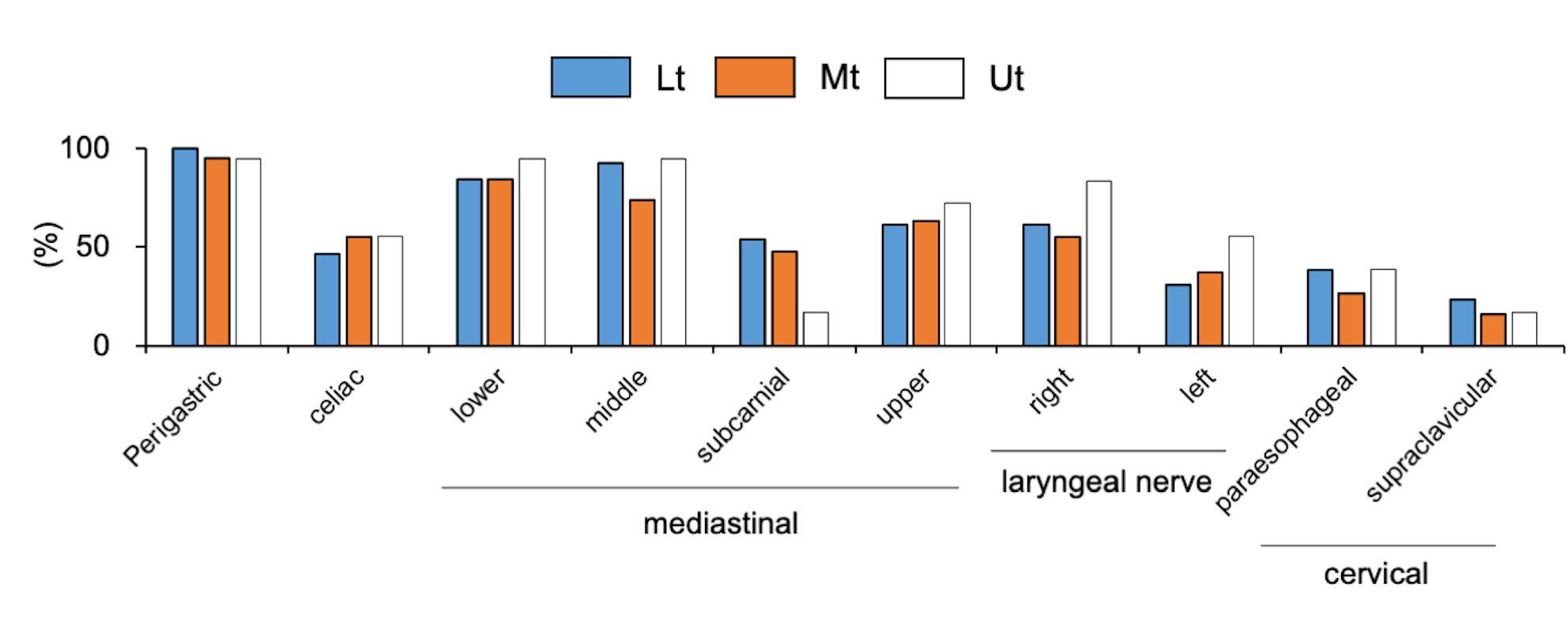

Supplement: Supplementary file 2 — Figure S2. The extent of lymphanedectomy according to tumor location. [file AGS3-9-952-s002.tiff]
