# Supplementary material for: Survival Outcomes of Esophageal Squamous Cell Carcinoma Patients Who Underwent Salvage Esophagectomy: A Literature Review and Results From Two High‐Volume Centers
Source: Ann Gastroenterol Surg. 2025 Apr 29;9(5):952–63. doi: 10.1002/ags3.70028 (PMC12414584; doi:10.1002/ags3.70028)
Supplement: Supplementary file 3 — Table S1. Node zones. [file AGS3-9-952-s001.docx]

| **Supplementary Table 1. Node Zones** | | | | |
| --- | --- | --- | --- | --- |
|  |  | Station number (JES) | Name of node station  (JES) | Station number  (AJCC) |
| Abdominal  Mediastinal  Lalyngeal nerve  Cervival | Perigastric  Celiac  Lower  Middle  subcarinal  Upper  Right  Left  Paraesophageal  Supraclavicular | 1  2  3  7  8  9  11  19, 20  110  111  112  108  107  109R/L  105  106recR  106recL  101R/L  104R/L | Right cardiac  Left cardiac  Lesser curvature  Left gastric artery  Common hepatic artery  Celiac  Splenic artery  Infradiaphragmatic  Lower paraesophageal  Supradiaphragmatic  Posterior mediastinum  Middle paraesophageal  Subcarinal  Right/Left main bronchus  Upper paraesophageal  Right recurrent nerve  Left recurrent nerve  Right/Left cervical paraesophageal  Right/Left supraclavicular | 16  16  17  18  20  19  8L  15  9  8M  7  10R/L  3p  2R  2L  1 |
| Abbreviations: JES, Japan Esophageal Society; AJCC, American Joint Committee on Cancer | | | | |
